# Bentonite as a Functional Material Enhancing Phytostabilization of Post-Industrial Contaminated Soils with Heavy Metals

**DOI:** 10.3390/ma15238331

**Published:** 2022-11-23

**Authors:** Barbara Klik, Jiri Holatko, Iwona Jaskulska, Mariusz Z. Gusiatin, Tereza Hammerschmiedt, Martin Brtnicky, Ernesta Liniauskienė, Tivadar Baltazar, Dariusz Jaskulski, Antonin Kintl, Maja Radziemska

**Affiliations:** 1 Institute of Environmental Engineering, Warsaw University of Life Sciences, 02-787 Warsaw, Poland; 2 Department of Agrochemistry, Soil Science, Microbiology and Plant Nutrition, Faculty of AgriSciences, Mendel University in Brno, Zemedelska 1, 613 00 Brno, Czech Republic; 3 Faculty of Agriculture and Biotechnology, Bydgoszcz University of Science and Technology, 85-796 Bydgoszcz, Poland; 4 Faculty of Geoengineering, University of Warmia and Mazury in Olsztyn, 10-719 Olsztyn, Poland; 5 Hydrotechnical Construction Department, Kaunas University of Applied Sciences, Liepu Str. 1, Girionys, LT-53101 Šlienava, Lithuania; 6 Agricultural Research, Ltd., Zahradni 1, 664 41 Troubsko, Czech Republic

**Keywords:** soil contamination, risk minimization, amendments, metal immobilization

## Abstract

Growing awareness of the risks posed by pollution of the soil environment is leading to the development of new remediation strategies. The technique of aided phytostabilization, which involves the evaluation of new heavy-metal (HM)-immobilizing amendments, together with appropriately selected plant species, is a challenge for environmental protection and remediation of the soil environment, and seems to be promising. In this study, the suitability of bentonite for the technique of aided phytostabilization of soils contaminated with high HM concentrations was determined, using a mixture of two grass species. The HM contents in the tested plants and in the soil were determined by flame atomic absorption spectrometry. The application of bentonite had a positive effect on the biomass of the tested plants, and resulted in an increase in soil pH. The concentrations of copper, nickel, cadmium, lead and chromium were higher in the roots than in the above-ground parts of the plants, especially when bentonite was applied to the soil. The addition of the analyzed soil additive contributed significantly to a decrease in the levels of zinc, copper, cadmium and nickel in the soil at the end of the experiment. In view of the above, it can be concluded that the use of bentonite in the aided phytostabilization of soils polluted with HMs, is appropriate.

## 1. Introduction

Substantial interference in the natural environment is a feature that is characteristic of dynamically developing industrialized countries. With the current rate of civilizational development and the increasingly frequent introduction of modern technologies, the pressures exerted on individual components of the natural environment, including the soil, continue to increase [1,2]. Due to the substantial impact of human activity on the environment, more attention is being paid to attempts to return degraded areas to an ecological balance, using various reclamation techniques. This particularly pertains to post-industrial areas, which are among some of the most heavily transformed and contaminated [3]. Soil contamination mainly concerns heavy metals (HMs), mineral oil and PAHs [4,5,6]. Among them, HMs pose the greatest threat, due to their prevalence, the number of contaminated sites, and their high toxicity [6,7]. However, in extensively polluted areas it is often difficult to identify the real risk of contamination [8], since the compounds of most HMs are easily soluble in the soil solution, and, thus highly assimilable by plants [9]. The mobility of HMs and their assimilability by plants is substantially influenced by soil conditions, including the content of organic matter, the pH, the sorption capacity, the form in which cations occur, the concentration of macro- and microelements, the oxidation-reduction potential, and the activity of microorganisms [10].

Hence the development of new environmentally-friendly reclamation technologies is amongst the most significant challenges of modern environmental engineering. So-called nature-based solutions, including aided phytostabilization, have been gaining popularity in recent years [11]. This technique relies on the effects that plants and various aiding substances have on the contaminants found in the soil, particularly HMs. Organic substances that are produced by plants and released into the rhizosphere, as well as soil amendments that are applied to the plants, can change the pH of the soil and influence its oxidation-reduction potential, in addition to reducing the ions of toxic HMs to forms that are unavailable to plants [12]. This process immobilizes HMs, limiting their ability to move deeper into the soil profile, to groundwater, and to subsequent links of the food chain [13]. Moreover, this is a low-cost technique, making it especially useful for the reclamation of large areas. It can be implemented on various types of degraded terrain, including urbanized or post-industrial areas [14].

Aided phytostabilization is among the most ecologically-friendly methods, as it does not involve the creation of waste dumps or the contamination of the environment resulting from transporting contaminated soil to another location. In order to limit the mobility of HMs and increase their immobilization in the soil environment, various types of soil additives (organic or mineral) are used [15]. These additives aim to create non-soluble HM complexes with limited availability, and to aid the regrowth of impoverished vegetation cover in contaminated areas [16]. The immobilization of HMs can be aided by the addition of organic matter in the form of compost or sewage sludge, or the addition of carbonates, phosphate minerals, or clay minerals [17,18].

Bentonites are mudstones consisting mainly of smectites, particularly montmorillonite, which is a hydrated aluminosilicate with a layered structure [19]. Their characteristic features include their substantial specific surface area and their tendency to adsorb water in interstitial spaces, as a result of which, bentonite has the greatest tendency to swell of all clay minerals (water absorption 300–700%) [20]. The composition of bentonites varies, due to isomorphic substitutions within Al^+3^ ion chains, for example, with Mg^+2^ and Fe^+3^ ions, and the predominant cation, i.e., Ca^+2^ or Na^+^ [21]. Some Al^3+^ ions are replaced with cations at a lower oxidation level, which generates a negative charge in the entire layer. This charge is compensated for by cations (Mg^2+^, Na^+^, Ca^2+^, K^+^) located in interstitial spaces. Other cations can activate ion exchange [22]. Bentonite is a valuable source of ingredients for plants, due to its high content of macro- and microelements, thus supporting the growth and development of biomass. The positive effect of bentonite on plants, manifested in an increase in yield, has also been confirmed by other authors [23,24].The addition of bentonite increases field-water holding-capacity and the water available to plants. It is a stable mineral that requires only a single application, and therefore has a distinct advantage over synthetic water-absorbing polymers that degrade over several years and must be periodically reapplied to maintain their effectiveness [25]. Considering the above, it can be concluded that the use of bentonite in the technique of assisted phytostabilization can not only support the immobilization of HMs in the soil, but also contribute to a better vegetation cover of areas covered by the contamination. On the other hand, the use of bentonite as a soil amendment should be carefully considered, as it can cause an excessive increase in soil pH, which can immobilize important nutrients in the soil. A useful characteristic of bentonites is their ability to sorb cations and organic substances, making them suitable for applications in environmental engineering, e.g., water, sewage, and soil purification [26,27,28]. However, according to the available literature sources, the use of bentonite in aided phytostabilization of soils polluted with HMs has not yet been tested. Therefore, the objective of this study was to determine the effect of bentonite application on aided phytostabilization, including the yield and chemical composition of the above-ground biomass and roots of the test plants, as well as the contents of HMs in the multi-HM-contaminated soils.

## 2. Materials and Methods

### 2.1. Characterization of Soil Used in the Assisted Phytostabilization

Soil derived from the area of steel disposal dumps located in northeastern Poland was used for the pot experiment. The soil collected for experiments of aided phytostabilization was characterized by the high concentration of HMs, such as copper, nickel, cadmium, lead, zinc and chromium, which deviated from the standards set out for Poland in regard to heavy-metal content in the near-surface layer of the terrain [29]. Therefore, the selected parameters of the soil are listed in Table 1.

Soil samples were collected at the surface, using a stainless-steel shovel. At each sampling point, 20 different samples were collected from different points, mixed, and treated as composite samples. All samples were then placed in clean polyethylene bags and transported to the laboratory. Samples were then air dried, sieved through a 2-mm sieve, and stored in a refrigerator at 4 °C. The methodology used for sampling and analysis is described by Radziemska et al. [3].

### 2.2. Bentonite-Assisted Phytostabilization Experiment Description

The experiment was performed as described by Radziemska et al. [3]. Polyethylene pots (5 kg) were used for the experiment. Each pot was filled with a combination of soil and bentonite at a concentration of 3% (*w*/*w*). Polluted soil was used as a control. Bentonite (Ekobent B, KERAMOST, Most, Czech Republic) with a humidity content of 5%, pH 9.0 (in KCl), grain 0–2 mm, and montmorillonite concentration 70%, was used. The oxide content was: 535 g SiO_2_/kg; 24 g Al_2_O_3_/kg; 2.5 g Na_2_O/kg; 7.5 g K_2_O/kg; 1 g P_2_O_5_/kg. The soil in the pots with bentonite was kept for two weeks (in the dark) to be homogeneous before planting. Then, 5 g seeds of *Lolium perenne* L. (cv. Nur) and *Festuca rubra* L. (cv. Aido) were sown in each pot. The phytostabilization experiment was conducted in a greenhouse with an average temperature of 26 ± 3 °C during the day (approx.14 h) and 16 ± 2 °C at night (approx. 10 h). The experiment lasted for 56 days. Subsequently, the plants were harvested, weighed and divided into shoots (above-ground fragments) and roots (thoroughly washed with tap water).

### 2.3. Analytical Methods of Soil Characterization

The soils were ground to obtain a more homogeneous soil material, separately for each pot, using a soil grinder (H-4199.5F, Humboldt Mfg. Co., Elgin, IL, USA) and dried only at room temperature. The soil pH was measured using a pH meter (HI 221, Hanna Instrument, Woonsocket, RI, USA) in a soil/water slurry (1:2.5 *w*/*v*). For HM (Cu, Ni, Cd, Pb, Zn and Cr) assessment in soils, the samples were digested in HCl, HNO_3_, and H_2_O_2_ in a Microwave Digestion system (MARSXpress, CEM Corporation, Matthews, NC, USA) and determined by using an atomic absorption spectrotometer with flame atomization (Varian, AA28OFS, Mulgrave, Australia). Reference material (CRM 142 R) was used to appraise the quality of the analysis, and acquired recoveries varied from 95% to 101%. Throughout the experiment, ultra-pure water (Milli-Q System, Merck Millipore, Merck KGaA, Darmstadt, Germany) of 18 MΩcm resistivity was used.

### 2.4. Analytical Methods of Plant Analyses

After the phytostabilization experiment, the particular parts of the plants (above-ground and roots) were separated from each vessel, and sorted. The mass of fresh above-ground parts was measured (each vessel severally), using a balance (Acculab ATL-623-V, Sartorius, Goettingen, Germany). Prior to chemical exploration, the plants were measured and ground using an analytical mill (Retsch type ZM300, Hann, Germany). For the determination of Cu, Ni, Cd, Pb, Zn and Cr in plant parts, 0.5 g of the powdered sample of shoots and roots was weighed and digested in concentrated HNO_3_ and 30% H_2_O_2,_ using a Microwave Digestion system (MARSXpress, CEM Corporation, Matthews, NC, USA) and assessed using an atomic absorption spectrometer with flame atomization (Varian, AA28OFS, Mulgrave, Australia). The values of LOD, LOQ and accuracy (AO) for the analyzed elements are presented in Table 2.

### 2.5. Statistical Analysis

For statistical calculations and graphics, we used the flexible, free R software environment, version 3.6.3., (the R Project for Statistical Computing) [30]. To determine if there were a statistically significant difference between the mean values of total copper, nickel, cadmium, lead, zinc, and chromium in the soil and the selected treatments (control, bentonite), or between the mean values of plant biomass and the selected treatments (control, bentonite), we used a one-way analysis of variance (ANOVA). To determine the statistically significant difference using ANOVA, we applied Tukey’s test and a “treatment contrast” to estimate the mean values for each treatment. All statistical analyses were performed at a 5% significance level. In addition, normality was tested using the Shapiro–Wilk normality test, and Bartlett’s test was used to test variance homogeneity. In addition, different types of diagnostic plots were used to examine residual behavior.

## 3. Results and Discussion

### 3.1. Soil pH and HMs Content after Application of Bentonite

**Figure 1:**
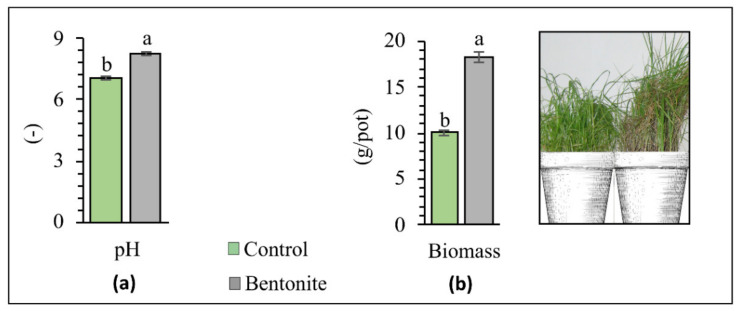
Soil pH (**a**) and plant biomass (**b**) after phytostabilization. Different letters indicate significant differences (*p* < 0.05) between the control samples and those to which bentonite was added.

Soil pH determines, among other effects, the uptake of HMs from the soil by plants and the degree of HM immobilization and/or mobilization [31]. In recent years, there has been a significant increase in the amount of acidic soils, caused by the introduction of industrial contaminants and the intensification of agricultural production [32,33]. Therefore, applying treatments aimed at increasing soil pH is considered one of the most effective types of remediation techniques. Bentonite contains large amounts of silicon dioxide, magnesium oxide, and calcium oxide, which reduce soil acidity [27]. The soil pH values at the time of the experiment’s completion are presented in Figure 1. The pH value was 1.21 higher in soils that received bentonite than in the control soils. It can therefore be concluded that bentonite possesses liming abilities, and thus decreases the exchangeable acidity and aluminum saturation in the soil [34].

**Figure 2:**
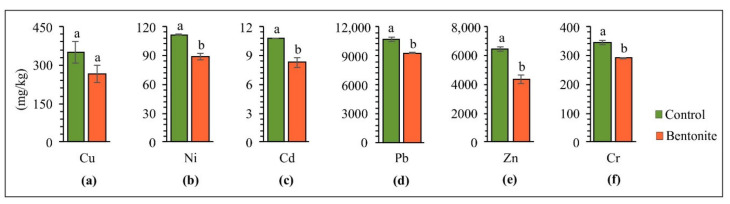
Soil HM composition after bentonite-assisted phytostabilization: (**a**) Cu, (**b**) Ni, (**c**) Cd, (**d**) Pb, (**e**) Zn, (**f**) Cr. Different letters indicate significant differences (*p* < 0.05) between the control and bentonite samples.

Figure 2 presents the total values of copper, nickel, cadmium, lead, zinc, and chromium in the soil upon completing the experiment of aided phytostabilization. The mobility and bioavailability of HMs in soil depend on physical and chemical factors as well as biological factors, and, above all, on the content of organic matter, biological activity, and pH [35]. Bentonite contains a high surface-area value, which leads to a high sorption ability in metal ions and the ability to trap HM ions in its structure, and enhance isomorphic substitution [36]. By introducing bentonite to HM-contaminated soil, significantly decreased total copper (24%), nickel (20%), cadmium (23%), lead (13%), zinc (32%) and chromium (16%) concentrations were observed in the soil, as compared to the control series. According to Hamidpour et al. [37], ion exchange is a dominant mechanism of cadmium sorption by bentonite. On the other hand, the irreversibility of lead sorption by soil amendments was due to the inner-sphere binding of lead to the mineral edges. In their work, Sun et al. [38] also showed a high level of cadmium and lead immobilization, indicating that bentonite possesses a high potential for stabilizing HMs in contaminated soils. The results of studies by Vrinceanu et al. [39] showed that, by introducing bentonite to the soil, the pH of the soil increased, thus leading to a more excellent retention of HMs in the solid-soil phase. The same author notices that the liming effect in this case leads to the binding of HMs by long-term diffusion into clay mineral layers. On the other hand, Zhang et al. [36], in a five-year experiment with *Capsicum annuum* L.–*Brassica pekinensis* L. rotation, reported that bentonite influenced a decrease in the leaching of cadmium and lead from the soil.

### 3.2. Plant Biomass after Application of Bentonite

Areas formed due to industrial activity are particular, and unfavorable to vegetation-cover growth. From the long-term perspective of applying the aided phytostabilization technique in soil contaminated with HMs, it is crucial to create an adequately dense vegetation-cover, which is usually very sparse in such areas [40]. Bentonite applied to the soil does not undergo degradation over time, and remains there, improving the retention of nutrients [41,42]. It can also be applied as a carrier of chemical fertilizers, which ensures a long-lasting fertilizing effect [43]. An evaluation of the results revealed that the test-plant yield significantly depends on the contamination of soil by HMs as well as the addition of bentonite (Figure 1b). In the control series (without bentonite), the above-ground parts of plants were relatively sensitive to soil contamination with HMs, and were found to have a significantly lower plant yield, as per the views of other authors. As mentioned, plant cover in areas where such high concentrations of HMs were found to occur is much sparser [44,45,46]. However, the application of bentonite significantly influenced an improvement and increase in the plant yield, which in this case was 39% higher. With the application of bentonite, the macronutrients were retained in the root zone, which significantly increased the ability of the roots to absorb more significant amounts of nutrients into plant tissues. This result is sustained by Youssef [47], who showed that an increased yield might result from an improvement in the soil’s physical and chemical properties (the availability of macronutrients), thanks to the application of bentonite. Mi et al. [26] also observed a significantly increased yield of *Triticum aestivum* L. after adding bentonite and nano bentonite. The results follow those given by Wafaa & Wagida [48], who revealed that the plant yield of *Triticum aestivum* L. significantly increased, thanks to the application of this soil amendment. Studies by Zhang et al. [36] showed that, compared to the control, the application of bentonite improved *Capsicum annuum* L. yields by an average of 29% during the 5-year study.

### 3.3. Content of HMs in Plants

**Figure 3:**
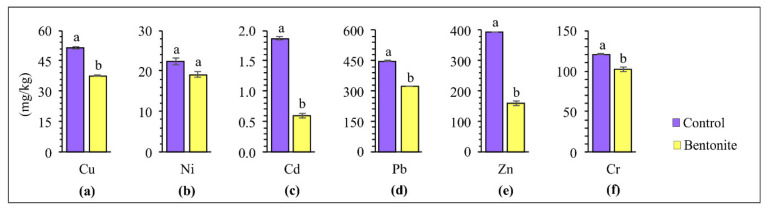
Content of copper (**a**), nickel (**b**), cadmium (**c**), lead (**d**), zinc (**e**), and chromium (**f**) in above-ground parts of tested plants. Different letters indicate significant differences (*p* < 0.05) between the control and bentonite samples.

**Figure 4:**
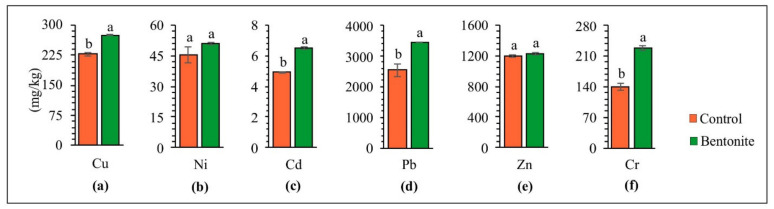
Content of copper (**a**), nickel (**b**), cadmium (**c**), lead (**d**), zinc (**e**), and chromium (**f**) in roots of tested plants. Different letters indicate significant differences (*p* < 0.05) between the control and bentonite samples.

Applying soil additives to the soil positively affected the soil’s physiochemical properties, thus alleviating the phytotoxic effect of HMs on plants [49]. The contents of copper, nickel, cadmium, lead, zinc, and chromium in the above-ground parts of the tested plants and roots after carrying out the studies with the application of bentonite-assisted phytostabilization are presented in Figure 3 and Figure 4. The concentration of HMs accumulated in the roots and above-ground parts depended on the type of metal and the applied bentonite. Research by other authors shows that grasses take up HMs rather intensively, and that the uptake of cadmium, lead, nickel and zinc by the roots generally increases under the influence of increasing contamination [50]. The higher content of all analyzed HMs was confirmed in the roots of plants, as compared to their above-ground parts, especially following the application of bentonite to the soil. This can be explained by the fact that bentonite may lead to the immobilization of HMs in soils, while at the same time improving their quality as well as significantly lowering the uptake of HMs by the above-ground parts of plants [36]. These differences were exceptionally high for lead. The content of Pb in the roots was ten times higher, whereas for copper and zinc was a maximum of 2–3 times higher than in the above-ground parts. On this basis, it can be assumed that the decrease in HM content in the above-ground parts of tested plants following the application of bentonites can be caused by an increase in the effectiveness of the immobilization and the decreased bioavailability of HMs [32]. Vrinceanu et al. [39] showed that bentonite significantly reduced the cadmium and zinc uptake in the above-ground parts of a mix of perennial grasses and straw cereals (*Trifolium pretense* L., *Dactylis glomerata* L., *Lolium perenne* L., *Agropyron repens* L.), from the soil. The table with ANOVA for HMs in soil, for above-ground parts and roots of plants, can be found in the Supplementary Materials (Table S1).

## 4. Conclusions

The impact of human activities on soil degradation can be observed almost everywhere, whether it is agricultural or industrial activities, or increasing urbanization. Activities related to soil conservation are responsible, among other things, for the development of new strategies for its remediation—one example is aided phytostabilization. In the present study, the influence of a new additive in the form of *Lolium perenne* L. and *Festuca rubra* L. on the immobilization of HMs was determined. After the experiment, the yield of the test plants in the pots to which bentonite had been added was 39% higher. This additive also contributed to a significant increase in soil pH (by 1.19 units) and, most significantly, reduced the total content of zinc (32%), copper (24%), cadmium (23%) and nickel (20%) in the soil, compared to the control series. Higher levels of copper, nickel, cadmium, lead, zinc, and chromium were observed in the roots of the tested plants than in the above-ground parts. Bentonite influenced an increase especially in the contents of chromium (39%), cadmium (32%) and copper (18%), in the roots. In conclusion, the application of bentonite can be an effective solution to support phytostabilization processes in soils contaminated by HMs.

## Figures and Tables

**Table 1:** Total HM concentration in the soil used in the bentonite-assisted phytostabilization.

Parameter	Unit	Value (Mean ± SD)	National Limit ^1^
Copper	mg/kg	681.0 ± 144	600
Nickel	mg/kg	1134 ± 24.5	300
Cadmium	mg/kg	21.31 ± 3.03	15
Lead	mg/kg	13.47 ± 642	600
Zinc	mg/kg	8433 ± 1.37	1000
Chromium	mg/kg	490.5 ± 62.3	500

^1^ Threshold concentration of HMs in soils according to the Polish Ministry of the Environment [29].

**Table 2:** LOD, LOQ and accuracy for the analyzed HMs.

Element	LOD (mg/L)	LOQ (mg/L)	Reference Value (mg/kg)	Measured Value (mg/kg)	AO (%)
Cu	0.008	0.027	46.9 ± 1.8	43.9 ± 2.3	93.6
Ni	0.056	0.171	94 ± 5.0	91.8 ± 1.7	97.6
Cd	0.023	0.075	14 ± 1.4	13.1 ± 1.6	93.6
Pb	0.020	0.060	51.3 ± 2.0	48.6 ± 2.4	94.7
Zn	0.066	0.220	270 ± 8.0	261 ± 6.4	96.7
Cr	0.072	0.219	138 ± 5.0	131 ± 3.1	94.9

## Data Availability

Not applicable.

## Supplementary Materials

The following supporting information can be downloaded at: https://www.mdpi.com/article/10.3390/ma15238331/s1, Table S1: ANOVA for HMs in soil, above-ground parts and roots of plants.

## References

[1] Díaz-Morales D.M., Erasmus J.H., Bosch S., Nachev M., Smit N.J., Zimmermann S., Wepener V., Sures B. (2021). Metal contamination and toxicity of soils and river sediments from the world’s largest platinum mining area. Environ. Pollut..

[2] Luo Y., Yuan H., Zhao J., Qi Y., Cao W.W., Liu J.M., Guo W., Bao Z.H. (2021). Multiple factors influence bacterial community diversity and composition in soils with rare earth element and heavy metal co-contamination. Ecotoxicol. Environ. Saf..

[3] Radziemska M., Gusiatin Z.M., Cydzik-Kwiatkowska A., Cerdà A., Pecina V., Bęś A., Datta R., Majewski G., Mazur Z., Dzięcioł J. (2021). Insight into metal immobilization and microbial community structure in soil from a steel disposal dump that was phytostabilized with composted, pyrolyzed or gasified wastes. Chemosphere.

[4] Panagos P., Van Liedekerke M., Yigini Y., Montanarella L. (2013). Contaminated sites in Europe: Review of the current situation based on data collected through a European network. J. Environ. Health.

[5] Boente C., Baragaño D., Gallego J.R. (2020). Benzo [a] pyrene sourcing and abundance in a coal region in transition reveals historical pollution, rendering soil screening levels impractical. Environ. Pollut..

[6] European Environment Agency (2019). The European Environment—State and Outlook 2020: Knowledge for Transition to a Sustainable Europe.

[7] Agency for Toxic Substances and Disease Registry Substance Priority List. https://www.atsdr.cdc.gov/spl/index.html#2019spl.

[8] Boente C., Gerassis S., Albuquerque M.T.D., Taboada J., Gallego J.R. (2020). Local versus regional soil screening levels to identify potentially polluted areas. Math. Geosci..

[9] Yan X., An J., Yin Y., Gao C., Wang B., Wei S. (2022). Heavy metals uptake and translocation of typical wetland plants and their ecological effects on the coastal soil of a contaminated bay in Northeast China. Sci. Total Environ..

[10] Thakur M., Praveen S., Divte P.R., Mitra R., Kumar M., Gupta C.K., Kalidindi U., Bansal R., Roy S., Anand A. (2022). Metal tolerance in plants: Molecular and physicochemical interface determines the “not so heavy effect” of heavy metals. Chemosphere.

[11] Lu X., Yamaji K., Haruma T., Yachi M., Doyama K., Tomiyama S. (2021). Metal Accumulation and Tolerance in *Artemisia indica* var. *maximowiczii* (Nakai) H. Hara. and *Fallopia sachalinensis* (F. Schmidt) *Ronse Decr*., a Naturally Growing Plant Species at Mine Site. Minerals.

[12] Chen Z.J., Tian W., Li Y.J., Sun L.N., Chen Y., Zhang H., Li Y.Y., Han H. (2021). Responses of rhizosphere bacterial communities, their functions and their network interactions to Cd stress under phytostabilization by *Miscanthus* spp.. Environ. Pollut..

[13] Peco J.D., Higueras P., Campos J.A., Esbrí J.M., Moreno M.M., Battaglia-Brunet F., Sandalio L.M. (2021). Abandoned Mine Lands Reclamation by Plant Remediation Technologies. Sustainability.

[14] Mazur Z., Radziemska M., Fronczyk J., Jeznach J. (2015). Heavy metal accumulation in bioindicators of pollution in urban areas of northeastern Poland. Fres. Environ. Bull..

[15] Labidi S., Firmin S., Verdin A., Bidar G., Laruelle F., Douay F., Shirali P., Fontaine J., Sahraoui A.L.H. (2017). Nature of fly ash amendments differently influences oxidative stress alleviation in four forest tree species and metal trace element phytostabilization in aged contaminated soil: A long-term field experiment. Ecotoxicol. Environ. Saf..

[16] Burges A., Epelde L., Benito G., Artetxe U., Becerril J.M., Garbisu C. (2016). Enhancement of ecosystem services during endophyte-assisted aided phytostabilization of metal contaminated mine soil. Sci. Total Environ..

[17] Scattolin M., Peuble S., Pereira F., Paran F., Moutte J., Menad N., Faure O. (2021). Aided-phytostabilization of steel slag dumps: The key-role of pH adjustment in decreasing chromium toxicity and improving manganese, phosphorus and zinc phytoavailability. J. Hazard. Mater..

[18] Lee S.H., Ji W.H., Lee W.S., Koo N., Koh I.H., Kim M.S., Park J.S. (2014). Influence of amendments and aided phytostabilization on metal availability and mobility in Pb/Zn mine tailings. J. Environ. Manag..

[19] Maanoja S., Palmroth M., Salminen L., Lehtinen L., Kokko M., Lakaniemi A.M., Auvinen H., Kiczka M., Muuri E., Rintala J. (2021). The effect of compaction and microbial activity on the quantity and release rate of water-soluble organic matter from bentonites. Appl. Clay Sci..

[20] Laufek F., Hanusová I., Svoboda J., Vašíček R., Najser J., Koubová M., Čurda M., Pticen F., Vaculíková L., Sun H. (2021). Mineralogical, geochemical and geotechnical study of BCV 2017 bentonite—The initial state and the state following thermal treatment at 200 °C. Minerals.

[21] Bobrowska M., Szaniawska D. (2011). Badania modyfikacji bentonitów do zastosowań w ochronie środowiska. Inż. Ap. Chem..

[22] Natkański P., Białas A., Kuśtrowski P. (2012). Synteza kompozytów poli(kwas akrylowy)-bentonit oraz poliakryloamid-bentonit do zastosowań adsorpcyjnych. Chemik.

[23] Kobus J. (1983). Wpływ nawożenia gleby piaskowej luźnej kaolinitem i bentonitem na plon roślin zbożowych i zawartość w nich niektórych składników mineralnych. Rocz. Glebozn..

[24] Rolka E. (2014). The yields of selected crops on soils contaminated by cadmium and supplied neutralizing substances. Zesz. Probl. Postępów Nauk. Rol..

[25] Mi J., Gregorich E.G., Xu S., McLaughlin N.B., Liu J. (2020). Effect of bentonite as a soil amendment on field water-holding capacity, and millet photosynthesis and grain quality. Sci. Rep..

[26] Mi J., Gregorich E.G., Xu S., McLaughlin N.B., Ma B., Liu J. (2021). Changes in soil biochemical properties following application of bentonite as a soil amendment. Eur. J. Soil Biol..

[27] Elmorsi R.R., Mostafa G.A.H., Abou-El-Sherbini K.S. (2021). Homoionic soda-activated bentonite for batch-mode removal of Pb(II) from polluted brackish water. J. Environ. Chem. Eng..

[28] Awasthi M.K., Awasthi S.K., Wang Q., Awasthi M.K., Zhao J., Chen H., Ren X., Wang M., Zhang Z. (2018). Role of Ca-bentonite to improve the humification, enzymatic activities, nutrient transformation and end product quality during sewage sludge composting. Bioresour. Technol..

[29] (2016). Ordinance of the Minister of Environment on soil and ground quality standards. J. Law.

[30] R Core Team (2019). R: A Language and Environment for Statistical Computing.

[31] Pérez-Esteban J., Escolástico C., Masaguer A., Vargas C., Moliner A. (2014). Soluble organic carbon and pH of organic amendments affect metal mobility and chemical speciation in mine soils. Chemosphere.

[32] Raza S., Zamanian K., Ullah S., Kuzyakov Y., Virto I., Zhou J. (2021). Inorganic carbon losses by soil acidification jeopardize global efforts on carbon sequestration and climate change mitigation. J. Clean. Prod..

[33] Anza M., Garbisu C., Salazar O., Epelde L., Alkorta I., Martínez-Santos M. (2021). Acidification alters the functionality of metal polluted soils. Appl. Soil Ecol..

[34] Tahervand S., Jalali M. (2017). Sorption and desorption of potentially toxic metals (Cd, Cu, Ni and Zn) by soil amended with bentonite, calcite and zeolite as a function of pH. J. Geochem. Explor..

[35] Tong H., Chen M., Lv Y., Liu C., Zheng C., Xia Y. (2021). Changes in the microbial community during microbial microaerophilic Fe(II) oxidation at circumneutral pH enriched from paddy soil. Environ. Geochem. Health.

[36] Zhang D., Ding A., Li T., Wu X., Liu Y., Naidu R. (2021). Immobilization of Cd and Pb in a contaminated acidic soil amended with hydroxyapatite, bentonite, and biochar. J. Soils Sediments.

[37] Hamidpour M., Kalbasi M., Afyuni M., Shariatmadari H., Holm P.E., Hansen G.C.B. (2010). Sorption hysteresis of Cd(II) and Pb(II) on natural zeolite and bentonite. J. Hazard. Mater..

[38] Sun Y., Li Y., Xu Y., Liang X., Wang L. (2015). In situ stabilization remediation of cadmium (Cd) and lead (Pb) co-contaminated paddy soil using bentonite. Appl. Clay Sci..

[39] Vrînceanu N.O., Motelică D.M., Dumitru M., Calciu I., Tănase V., Preda M. (2019). Assessment of using bentonite, dolomite, natural zeolite and manure for the immobilization of heavy metals in a contaminated soil: The Copșa Mică case study (Romania). Catena.

[40] Zine H., Midhat L., Hakkou R., Adnani M., Ouhammou A. (2020). Guidelines for a phytomanagement plan by the phytostabilization of mining wastes. Sci. Afr..

[41] Datta R., Holatko J., Latal O., Hammerschmiedt T., Elbl J., Pecina V., Kintl A., Balakova L., Radziemska M., Baltazar T. (2020). Bentonite-Based Organic Amendment Enriches Microbial Activity in Agricultural Soils. Land.

[42] Hermida L., Agustian J. (2019). Slow release urea fertilizer synthesized through recrystallization of urea incorporating natural bentonite using various binders. Environ. Technol. Innov..

[43] Sarkar A., Biswas D.R., Datta S.C., Dwivedi B.S., Bhattacharyya R., Kumar R., Bandyopadhyay K.K., Saha M., Chawla G., Saha J.K. (2021). Preparation of novel biodegradable starch/poly(vinyl alcohol)/bentonite grafted polymeric films for fertilizer encapsulation. Carbohydr. Polym..

[44] Pérez-de-Mora A., Madrid F., Cabrera F., Madejón E. (2007). Amendments and plant cover influence on trace element pools in a contaminated soil. Geoderma.

[45] Massenet A., Bonet A., Laur J., Labrecque M. (2021). Co-planting *Brassica napus* and *Salix nigra* as a phytomanagement alternative for copper contaminated soil. Chemosphere.

[46] Morosini C., Terzaghi E., Raspa G., Zanardini E., Anelli S., Armiraglio S., Petranich E., Covelli S.M., Guardo A. (2021). Mercury vertical and horizontal concentrations in agricultural soils of a historically contaminated site: Role of soil properties, chemical loading, and cultivated plant species in driving its mobility. Environ. Pollut..

[47] Youssef S.B.D. (2013). Effect of bentonite and zeolite ores on potato crop (*Solanum tuberosum* L.) under north Sinai conditions. J. Plant Prod..

[48] Wafaa M.T.E., Wagida Z.H. (2017). Effect of potassium humate and bentonite on some soil chemical properties under different rates of nitrogen fertilization. J. Soil Sci. Agric. Eng..

[49] Zhou H., Zhou X., Zeng M., Liao B.H., Liu L., Yang W.T., Wu Y.M., Qiu Q.Y., Wang Y. (2014). Effects of combined amendments on heavy metal accumulation in rice (*Oryza sativa* L.) planted on contaminated paddy soil. Ecotoxicol. Environ. Saf..

[50] Roongtanakiat N., Chairoj P. (2001). Uptake potential of some heavy metals by vetiver grass. Kasetsart. J. Nat. Sci..

