# Supplementary material for: Bentonite as a Functional Material Enhancing Phytostabilization of Post-Industrial Contaminated Soils with Heavy Metals
Source: Materials (Basel). 2022 Nov 23;15(23):8331. doi: 10.3390/ma15238331 (PMC9735557; doi:10.3390/ma15238331)
Supplement: Supplementary file 1 [file materials-15-08331-s001.zip › materials-2022074-supplementary.pdf]

Table S1. ANOVA for HMs in soil, above-ground parts and roots of plants

**Soil after phytostabilization**							
	**Locality 1 (control)**	**Locality 2 (control)**	**Locality 3 (bentonite)**	**Locality 4 (bentonite)**	**d.f.**	**F**	**p**
Mean Cu (mg/kg)	306.4	392.1	231.3	301.1	1,2	2.26	0.28
Mean Ni (mg/kg)	110.7	111.9	85.5	92.4	1,2	41.26	0.02*
Mean Cd (mg/kg)	10.8	10.8	7.8	8.8	1,2	24.70	0.04*
Mean Pb (mg/kg)	10895.2	10494.1	9248.8	9327.3	1,2	47.37	0.02*
Mean Zn (mg/kg)	6604.0	6277.6	4633.4	4076.2	1,2	41.74	0.01*
Mean Cr (mg/kg)	335.1	353.1	294.0	287.5	1,2	31.25	0.03*
**Above-ground parts**							
	**Locality 1 (control)**	**Locality 2 (control)**	**Locality 3 (bentonite)**	**Locality 4 (bentonite)**	**d.f.**	**F**	**p**
Mean Cu (mg/kg)	51.1	52.2	38.2	37.2	1,2	341.64	0.003**
Mean Ni (mg/kg)	21.5	23.2	20.0	18.3	1,2	7.51	0.11
Mean Cd (mg/kg)	1.9	1.9	0.6	0.6	1,2	883.61	0.001**
Mean Pb (mg/kg)	449.6	445.7	320.9	323.2	1,2	2967.9	<0.001***
Mean Zn (mg/kg)	394.3	395.2	152.5	165.4	1,2	1339.2	<0.001***
Mean Cr (mg/kg)	120.2	121.2	105.6	99.2	1,2	31.59	0.03*
**Roots**							
	**Locality 1 (control)**	**Locality 2 (control)**	**Locality 3 (bentonite)**	**Locality 4 (bentonite)**	**d.f.**	**F**	**p**
Mean Cu (mg/kg)	232.5	222.5	276.7	273.2	1,2	80.74	0.01*
Mean Ni (mg/kg)	49.6	41.4	50.2	51.3	1,2	1.65	0.33
Mean Cd (mg/kg)	4.9	4.9	6.5	6.5	1,2	1257.5	<0.001***
Mean Pb (mg/kg)	2333.2	2755.6	3461.5	3451.2	1,2	16600	0.049*
Mean Zn (mg/kg)	1186.6	1211.6	1217.1	1245.2	1,2	956.65	0.23
Mean Cr (mg/kg)	130.9	147.7	223.9	232.2	1,2	761.22	0.01*

* statistically significant difference at 0.05 significance level, ** statistically significant difference at 0.01 significance level, *** statistically significant difference at 0.001 significance level; d.f. = degrees of freedom (in ANOVA analysis)
